# Survival following psychiatric diagnoses in early adulthood

**DOI:** 10.1177/00048674251332562

**Published:** 2025-04-15

**Authors:** Kim S Betts, Rosa Alati, Peter M McEvoy, Daniel Rock, Kevin EK Chai, Crystal Man Ying Lee, Suzanne Robinson

**Affiliations:** 1 School of Population Health, Curtin University, Perth, WA, Australia; 2 Centre for Clinical Interventions, North Metropolitan Health Service, Perth, WA, Australia; 3 WA Primary Health Alliance, Perth, WA, Australia; 4 Discipline of Psychiatry, Medical School, The University of Western Australia, Perth, WA, Australia; 5 Faculty of Health, University of Canberra, Canberra, ACT, Australia; 6 Deakin Health Economics, Deakin University, Melbourne, VIC, Australia

**Keywords:** Mortality, inpatient admission, psychiatric diagnosis, early adulthood

## Abstract

**Aims:**

To establish the increased all-cause mortality risk after an inpatient episode of care with a diagnosis of a severe psychiatric disorder in young people.

**Methods:**

The data included all psychiatric inpatient episodes for psychiatric diagnoses in Western Australia between 2005 and 2022 linked with the state death registry. Participants were only included if they turned 18 years of age between 2005 and 2016, so survival from first adult admission until the study end date could be compared with age-gender matched life tables.

**Results:**

A total of 18,893 individuals had an admission with a primary or secondary diagnosis for a selected psychiatric diagnosis in the study period, across which time 485 died. Admission for substance use disorders presented the greatest risk of mortality, increasing the risk of death in early adulthood by more than three times (observed/expected = 3.07; 95% confidence interval = [2.76, 3.42]; *p* < 0.001), followed closely by bipolar disorders (observed/expected = 2.95; 95% confidence interval = [2.09, 4.03]; *p* < 0.001), while having any two or more comorbid disorders was associated with an increased death rate (observed/expected = 3.30; 95% confidence interval = [2.72, 3.97]; *p* < 0.001). The Kaplan–Meier curves also suggested that the proportionate increased risk of mortality remained relatively constant across the study period for all diagnoses.

**Conclusion:**

Inpatient admission for psychiatric disorders increased the risk of all-cause mortality in early adulthood by between two and three times and the increased death rate did not substantively reduce over time. Effective long-term support services are needed to reduce the premature mortality observed among these young adults.

## Introduction

It is now well established that individuals with mental health problems are at an increased risk of premature mortality (Ali et al., 2022; Chan et al., 2023; Walker et al., 2015). A number of factors have been identified as contributors to death among this population, including heart disease, infections, accidents, drug overdose and suicide (Plana-Ripoll et al., 2019; Walker et al., 2015). The mortality gap between people with and without psychiatric disorders is now understood as an important source of health inequality and an international health priority (Firth et al., 2019; O’Connor et al., 2023). Importantly, the increase in mortality is found to differ markedly across the various psychiatric diagnosis, with substance use disorders (SUD) consistently being associated with the highest increase, followed by eating disorders (Chan et al., 2023; Chesney et al., 2014; Plana-Ripoll et al., 2019), and with higher risks associated with combinations of psychiatric disorders (Plana-Ripoll et al., 2020) and psychiatric disorders accompanied by comorbid physical illness (Momen et al., 2022).

The magnitude of the increased mortality risk also depends on the severity of the disorder, which because most data within the literature is drawn from jurisdictional administrative data collections, is often measured by proxy as type of service usage. For example, multiple studies have found higher mortality rates associated with inpatient admission compared with outpatient or community patients (Crump et al., 2013; Musgrove et al., 2022; Walker et al., 2015). Due to differences in service availability and referral patterns across countries, in addition to other methodological differences such as the age group under study (Ali et al., 2022; Chan et al., 2023), it is difficult to determine the scale of the problem in many specific settings.

This is particularly true of the Australian setting, which is underrepresented in the current literature. In the most recent meta-analysis investigating excess mortality across a broad range of psychiatric diagnosis, only 6 of the total 109 studies were Australian (Chan et al., 2023). Of these, three specifically investigated mortality rates and causes among opioid users (Degenhardt et al., 2014; Lewer et al., 2020) and users of other substances (Tisdale et al., 2021), one focused on elderly subjects (Almeida et al., 2014), and one assessed the risk of cardiometabolic mortality in people with schizophrenia (Castle and Chung, 2018). The only Australian study to measure the increased risk of mortality among a broad range of psychiatric diagnoses was conducted in Western Australia (WA) and covered a period between 1985 and 2005, finding the disparity in life expectancy between those with and without psychiatric disorder increased over this 20-year period (Lawrence et al., 2013). Thus, a re-examination of this disparity in a contemporary WA cohort is well overdue.

In this study, we used WA inpatient and death registry data between 2005 and 2021 to derive population-based estimates of the increased mortality associated with specific psychiatric diagnoses. We focused specifically on early adulthood, such that all subjects enter adulthood (i.e. turn age 18) during the study period, which allows us to accurately measure the increased risk of mortality from first-onset adult diagnosis. Furthermore, we only considered diagnoses which resulted from an inpatient hospital stay (public and private hospitals), because administrative data collections contain complete coverage of these cases, while only partially capturing diagnoses resulting from community treatment.

## Methods

### Sample

The sample included all individuals in the State of WA who turned 18 years of age between January 2005 and December 2016, and who were admitted for an inpatient hospital stay (public or private hospital) within this time frame for an index ICD-10 psychiatric disorder (primary or secondary diagnosis) recorded in the Hospital Morbidity Data Collection. This data was linked to the WA Death Registrations between January 2005 and September 2022, such that all subjects were followed for between 18 and 5 years since turning 18. This resulted in a total of 18,893 individuals with records of one or more inpatient episodes for a psychiatric disorder during early adulthood. The linked data were sourced from administrative health data systems held by the WA Department of Health and the death register held by the Department of Justice, with further information found elsewhere (Lee et al., 2024). Linkage was carried out by the Western Australian Data Linkage Branch using probabilistic record linkage methods (Hodges et al., 2019). Ethics approval was granted by the Department of Health Western Australia Human Research Ethics Committee (approval number: RGS0000004782) and the Curtin University Human Research Ethics Committee (approval number: HRE2022-0001).

### Psychiatric diagnoses

Psychiatric diagnoses were taken from inpatient records contained in the WA Hospital Morbidity Data Collection (HMDC) which includes record level data related to episodes of care of patients admitted to both public and private acute hospitals in WA. Each episode of care includes one primary diagnosis and one co-diagnosis, for which one or both must be for a selected psychiatric disorder to be included in the study (with the primary diagnosis given precedence if both were psychiatric disorders). The diagnoses were separated into a number of groups according to ICD-10 codes, including substance use disorders (SUD) [ICD 10: F10 to F19.9], Psychotic disorders [ICD 10: F20 to F29], Bipolar/manic disorders (BPD) [ICD 10: F30 to F31.9], depressive disorders (DEP) [ICD 10: F32 to F39] and generalised anxiety disorders (GAD) [ICD 10: F41.1].

### Data analyses

Kaplan–Meier curves and one-sample log-rank tests were used to compare the observed survival among the patient sample with the expected survival among a standard population following the approach outlined by Finkelstein et al. (2003). This analysis was carried out separately for each disorder group and for co-occurrence, such that a patient entered the risk set on the date they were first admitted for the given disorder and was followed until death or the end of the observation period (i.e. censoring) regardless of whether the patient received additional diagnoses in the interim. If a patient did receive a diagnosis at a later date for another disorder, they then entered the risk set for that disorder on that date also (thus for each additional diagnoses the patient was treated as an independent observation).

Once in a risk set, the patient’s observed survival was compared with the expected survival obtained from Australian life tables made available by the Australian Bureau of Statistics (ABS) (2023b), matched by age (at first diagnosis), gender and year of birth. For example, if a male patient born in 1990 was first admitted for depression as an adult at age 20, his survival was compared with the average survival for all Australian males born in 1990 beginning at age 20. In this way, an expected death rate was obtained for each patient’s observation period, which when summed across all patients equalled the number of deaths that would be expected in an age/gender/year matched sample in the reference population. The ratio of deaths observed [*O*] in the sample versus expected deaths [*E*] in the matched reference population indicates the excess death experienced by the patient sample. The test for equality of mortality is obtained by the formula [*O – E*]^2^*/E*, for which the null hypothesis is distributed chi-square with 1 degree of freedom (Finkelstein et al., 2003).

To produce the survival curve for the reference population, the expected death rate was obtained for each patient as described above, but this time each patient’s death rate was calculated cumulatively at each age greater than their first admission for as many years as the longest length of follow-up. Individual expected cumulative death rates were then converted to expected survival rates using the formula given in Finkelstein et al. (2003). These were then summed to give the expected survival curve for the age/gender/year matched sample which was plotted against the observed survival curve. All analyses were undertaken in the R statistical package.

## Results

Table 1 shows the age and year of first adult admission for the selected psychiatric diagnosis groups among the total 18,893 individuals. The table shows that within each year, the frequency of first adult admissions is higher at younger ages, while within age groups no distinct trend is observed. The median age of first admission was 23 years (25th–75th percentile = 20–26 years) and the median length of follow-up (i.e. time from first admission to death or censoring) was 6.6 (25th–75th percentile = 3.7–10.0) years. In total, 485 (2.6%) individuals died during the study period at a median age of 26.7 years (25th–75th percentile = 23.5–29.9). Finally, 15,703 (83%) individuals had one or more admissions for a single recorded diagnosis only, 2732 (14%) had multiple admissions for two different recorded psychiatric diagnoses, while the remaining 497 (3%) had multiple admissions for three or four different recorded psychiatric diagnoses.

**Table 1.** Distribution of age and year at first adult admission for the selected psychiatric disorders (*n* = 19,250).

Year/age	18	19	20	21	22	23	24	25	26	27	28	29	30	31	32	33	34	35
2005	64	0	0	0	0	0	0	0	0	0	0	0	0	0	0	0	0	0
2006	146	135	0	0	0	0	0	0	0	0	0	0	0	0	0	0	0	0
2007	147	129	122	0	0	0	0	0	0	0	0	0	0	0	0	0	0	0
2008	122	135	116	111	0	0	0	0	0	0	0	0	0	0	0	0	0	0
2009	130	157	144	129	107	0	0	0	0	0	0	0	0	0	0	0	0	0
2010	175	175	142	138	123	137	0	0	0	0	0	0	0	0	0	0	0	0
2011	200	159	154	136	149	132	132	0	0	0	0	0	0	0	0	0	0	0
2012	220	207	175	182	158	151	137	140	0	0	0	0	0	0	0	0	0	0
2013	202	136	140	161	133	160	128	124	115	0	0	0	0	0	0	0	0	0
2014	182	155	148	139	151	129	128	120	119	109	0	0	0	0	0	0	0	0
2015	170	177	156	142	145	124	134	119	132	138	145	0	0	0	0	0	0	0
2016	187	164	167	162	153	127	125	127	133	130	95	129	0	0	0	0	0	0
2017	0	187	179	156	152	126	109	128	112	111	112	102	126	0	0	0	0	0
2018	0	0	154	142	148	152	128	101	116	100	109	132	102	126	0	0	0	0
2019	0	0	0	140	164	150	108	123	135	109	115	118	117	138	121	0	0	0
2020	0	0	0	0	116	156	120	110	116	96	95	111	96	111	102	104	0	0
2021	0	0	0	0	0	121	119	112	116	115	112	106	111	96	117	101	106	0
2022	0	0	0	0	0	0	31	18	29	30	25	21	31	27	22	22	24	24

**Figure 1.**
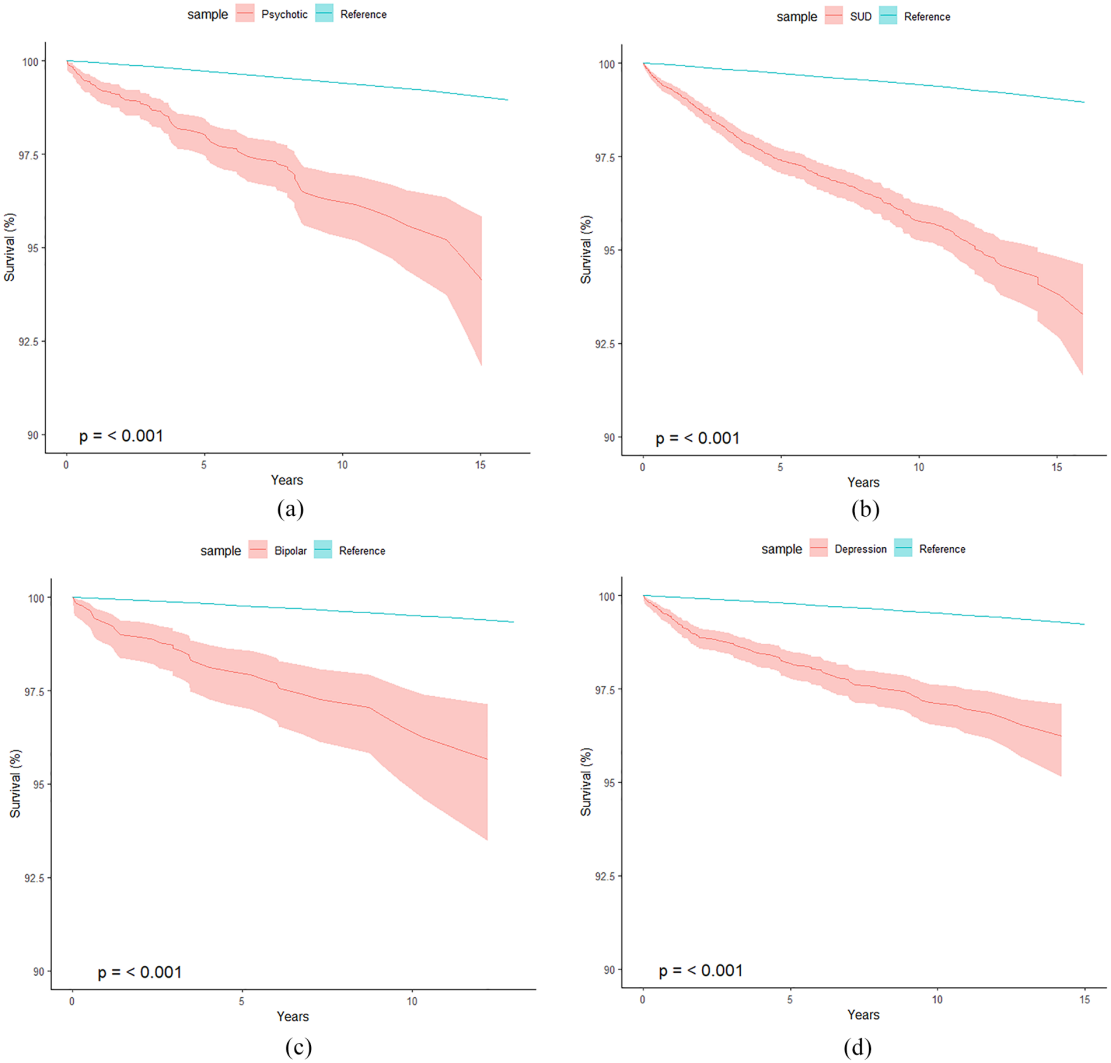
(a) Survival curve of all-cause mortality among those diagnosed with psychotic disorders in an inpatient episode of care versus the reference population (*n* = 3754, total years of life since DX = 25,375, observed deaths = 98, expected deaths = 46.5). (b) Survival curve of all-cause mortality among those diagnosed with SUD in an inpatient episode of care versus the reference population (*n* = 10,714, total years of life since SUD DX = 75,958, observed deaths = 345, expected deaths = 112.2). (c) Survival curve of all-cause mortality among those diagnosed with bipolar in an inpatient episode of care versus the reference population (*n* = 1658, total years of life since BPD DX = 10,588, observed deaths = 39, expected deaths = 13.2) and (d) Survival curve of all-cause mortality among those diagnosed with depressive disorders in an inpatient episode of care versus the reference population (*n* = 6454, total years of life since DEP DX = 42,905, observed deaths = 135, expected deaths = 58.9). Y-axis ranges from 100% to 90% survival.

Figure 1(a)–(d) shows the survival curves for each psychiatric disorder group (separate analyses) versus the reference population, with all four disorders showing a statistically significant increased risk of mortality compared with the reference population. SUD had the highest risk of mortality with 345 deaths among the 10,714 individuals, which was more than 3 times the 112 deaths expected. Furthermore, there was no obvious change in the proportionate mortality increase across time from first adult admission to the maximum follow-up of 15 years. BPD had the next highest mortality rate, with individuals at 2.95 (95% CI = [2.09, 4.03]) times the risk of mortality compared with the reference sample in the years following their first adult admission for BPD. Finally, DEP and psychotic disorders were associated with a 2.29 (95% CI = [1.92, 2.71]) and 2.11 (95% CI = [1.71, 2.57]) times increased risk of mortality compared with the reference populations.

**Figure 2.**
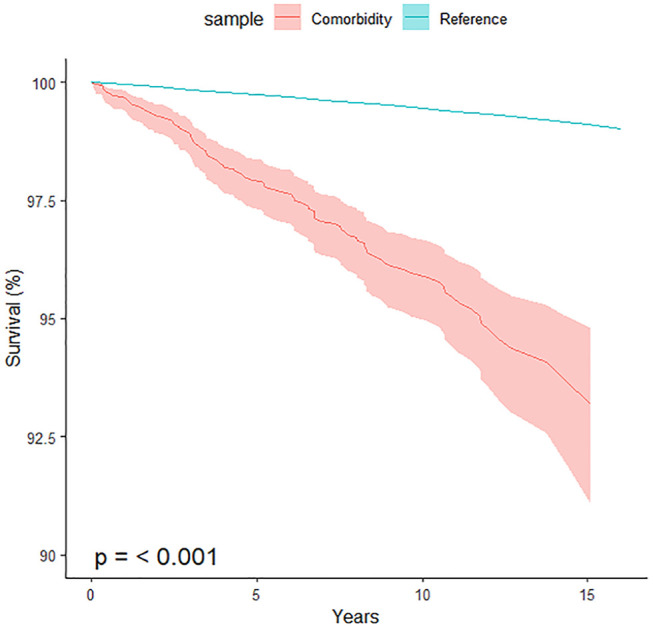
Survival curve of all-cause mortality among those diagnosed with two or more different disorders in two or more inpatient episode of care versus the reference population (*n* = 3190, total years of life since the first diagnosis DX = 26,364, observed deaths = 112, expected deaths = 33.9).

Figure 2 shows the survival curve for those who had two or more different diagnoses (i.e. comorbid disorders) from two or more admissions compared with the reference population (with observation time beginning with the first admission). Individuals with a comorbid diagnosis (*n* = 3190) had 3.30 (95% CI = [2.72, 3.97]) times the risk of death over the period of observation compared with the reference population. Among this group, 78% (2489/3190) had an admission for SUD, 61% (1937/3190) for a psychotic disorder, 49% (1560/3190) for DEP and 28% (891/3190) for BPD. SUD and Psychotic disorders were the most common comorbid disorders (1460/3190 = 46%), followed by SUD and DEP (1050/3190 = 33%). Importantly, sample size limitations prevented us from examining further aspects of co-occurrence (such as first-onset disorder and time elapsed between diagnoses).

## Discussion

In this study, we have provided up-to-date estimates of the increased mortality associated with psychiatric disorders requiring inpatient admission in a population of young adult Australians, indicating an increased risk of premature death of more than two and three times depending on the diagnosis. In line with comparable studies, we found that SUD (Chan et al., 2023; Chesney et al., 2014; Plana-Ripoll et al., 2019) and co-occurrence (Plana-Ripoll et al., 2020) presented the greatest increased risk of mortality, with both associated with more than 3 times the death rate across the observation period. Furthermore, 78% of those with a comorbid diagnosis had an admission for SUD and SUD was also by far the most prevalent disorder in the full sample, indicating the dominance of this disorder in driving an increased risk of mortality among young adults.

The most recent meta-analysis on this topic by Chan et al. (2023) calculated the years-of-potential-life-lost (YPLL) by each psychiatric diagnosis among a pooled sample of more than 12 million patients (Chan et al., 2023). The authors found that SUD had the greatest YPLL of more than 20 years, which was 5 years higher than schizophrenia-spectrum disorders. This was in line with findings from one of the largest studies to date, utilising a Danish population of more than 7 million, which found that SUD had the highest mortality rate of all common psychiatric disorders (Plana-Ripoll et al., 2019). Furthermore, a recent study estimating the increased risk of mortality among common combinations of psychiatric disorders identified the combination of SUD with Schizophrenia and anxiety as conferring the highest risk (Plana-Ripoll et al., 2020). Thus, clinical interventions aimed at reducing premature mortality among SUD will be more effective if targeting specific substances, risky methods of using and contact with the criminal justice system.

Although our data did not include information regarding cause of death, research indicates that young adults with SUD are at risk of premature death from a number of causes, which partly depend on the substance used, including overdose, intoxication death, suicide, and severe somatic disorders such as liver and heart disease (Fridell et al., 2019; Hjemsæter et al., 2019; Kinner et al., 2015). The largest study to compare the cause of death among different psychiatric diagnoses found that SUD had among the highest rates of death due to accidents, suicide and homicide compared with other disorders (Plana-Ripoll et al., 2019). A later study by the same team found that SUD had a far greater relationship with the development of gastrointestinal and hematologic disorders compared with other disorders (Momen et al., 2020).

Our findings suggest that any research, health policy or programme aiming to reduce mortality among young adults with severe psychiatric disorders will need to include a strong focus on SUD. Although we did not have the statistical power to assess SUD mortality by substance type, previous research shows that some substances and substance users are at elevated risk. Fridell et al. (2019) followed 1405 patients with SUD across 42 years, finding that the risk of death by overdose or intoxication was much higher in opioid and alcohol users respectively, when compared with cannabis or amphetamine users (Fridell et al., 2019). Further studies have shown that alcohol use disorders had the highest rates of mortality due to somatic disease (Hjemsæter et al., 2019), and that young people who have served custodial sentences and inject drugs are at heightened risk of premature mortality (Hjemsæter et al., 2019).

Although our data indicates that SUD plays a key role in premature mortality among young people, it is important to note that the other psychiatric disorders in our analyses were also associated with at least a doubling in mortality. Despite their being far fewer patients with bipolar disorder compared with SUD (1658 vs 10,714), the increased mortality was statistically the same in both disorders (around three times), suggesting this disorder may also be a primary target for reducing mortality. However, we note that in the meta-analysis by Chan et al. (2023), the increased mortality of bipolar disorder was statistically less and the same as psychotic disorders and depressive disorders, respectively. This may either be due to methodological differences, in that we followed young people across time, or we may have produced a less reliable estimate of bipolar survival compared with the other conditions due to having relatively fewer patients with bipolar disorder.

Our unique method of constructing Kaplan–Meier curves of time to death for each diagnostic grouping versus a reference population led to an important novel observation concerning the risk of mortality across time. For all disorders, the proportionate increase in the death rate was relatively constant across time, from the individual’s first adult admission until the maximum follow-up of ~15 years. This has implications for the type and timing of support services made available to patients, as there does not appear to be a time since first adult admission in which the risk of mortality is greater or less. This likely reflects the range of different causes of death, from acute deaths such as overdose, intoxication and accidents to deaths associated with chronic conditions resulting from the psychiatric disorder. Hence, further research is needed to establish the timing of different causes of death among young adults with psychiatric disorders to help inform future support services.

Importantly, we excluded a number of common psychiatric disorders from our analysis, due to having a low prevalence among our inpatient sample. As two examples, only 667 and 559 individuals were admitted for a primary or secondary generalised anxiety disorder [ICD 10: F41.1] or eating disorders [ICD 10: F50-F50.9], for which fewer than 10 individuals died in each group. The low numbers likely reflect the fact that these disorders are less likely to require admission as part of the management strategy, meaning our data may miss a substantial proportion of the affected population. However, previous studies have found increased death rates among the fuller range of psychiatric disorders including anxiety, eating, personality and behavioural disorders (Chan et al., 2023), with eating disorders in particular found to be associated with a substantial increased mortality risk in certain populations (Van Hoeken and Hoek 2020).

Before considering the strengths and limitations of our analysis, it is worth briefly pointing out that we expect the COVID-19 pandemic to have had a relatively small impact on our findings. Although the study period included the pandemic, the study population was drawn entirely from the Australian state of WA. Due to its geographic isolation and pandemic border closures, WA was relatively unaffected by the COVID-19 pandemic, registering only 919 deaths due to COVID as of July 2023 (ABS, 2023a), despite having a population of 2.6 million (roughly 10% of the Australian population). Furthermore, the impacts of lockdowns and health service unavailability due to the pandemic were relatively mild and short-lived, as the Government opted to instead close the State border to international and national travellers.

Our study had a number of strengths, one of which was the novel method we used to measure mortality since the first adult admission, in addition to having data on all admissions from the age of 18 for up to 15 years. However, it is important to note that our data includes several limitations, which may have led to either underestimation or overestimation of mortality after first adult admission for severe psychiatric disorder. First, survivorship bias is likely to have reduced the mortality rates we observed. This occurs because some individuals with psychiatric disorders will die prior to being admitted, and thus our estimate is based on those who survived long enough to first be admitted and diagnosed prior to death. Second, our mortality rates may be further downward biased by incomplete follow-up. This would occur if a young adult left WA after having been admitted and diagnosed, but later died in another jurisdiction, as this individual would not be recorded as having died according to the WA death registry. On the other hand, our analysis assumes that the psychiatric diagnosis preceded any physical health problems which may have played a role in premature mortality. Although this is the likely scenario among young adults, it is still possible that some of the individuals in our data developed psychiatric disorders after, or in response to, developing a life-limiting physical disorder, resulting in an upwards biased mortality estimate.

In conclusion, we found a substantial increased risk of mortality among young adult Australians in the years following their first adult admission for a severe psychiatric disorder. We hope these findings are utilised to justify and assist the creation and implementation of programmes and policies aiming to reduce mortality among this population. Further research is also needed to identify sub-groups at the greatest risk of mortality, in addition to the associated factors and timing of death.
